# Guided by the northern star coral: a research synthesis and roadmap for *Astrangia poculata*

**DOI:** 10.1098/rsbl.2024.0469

**Published:** 2025-03-19

**Authors:** Jill Ashey, Hollie M. Putnam, M. Conor McManus

**Affiliations:** ^1^ University of Rhode Island, Kingston, RI, USA; ^2^ Division of Marine Fisheries, Rhode Island Department of Environmental Management, Jamestown, RI, USA

**Keywords:** *Astrangia poculata*, coral, symbiosis, life history

## Abstract

The northern star coral, *Astrangia poculata*, is a temperate, facultatively symbiotic, scleractinian coral spanning the coastal western Atlantic. This calcifying species is mixotrophic with a broad geographical range, and therefore has high utility in addressing questions related to community ecology, symbiosis, population genetics, biomineralization and resilience to environmental perturbations. Here, we review the current *A. poculata* peer-reviewed literature, which is primarily found in six focal areas: geographic range, habitat and ecology, symbiosis, life history, microbiome and genomics and transcriptomics. A cross-cutting theme of these studies emerges as the value of an experimental system that is facultatively symbiotic. Yet, the historic overgeneralization of symbiotic versus ‘aposymbiotic’ *A. poculata* has constrained the interpretation of the basic biology and generalizability of conclusions. Emergent from our review, and timely with respect to climate change, is the value that *A. poculata* brings as an experimental system with the potential to test questions on range adaptability and environmental resilience. We identify future avenues of research for *A. poculata* studies that include integration of population genetics with organismal–molecular–cellular biology across the geographical range, while leveraging the power of the facultative symbiosis context.

## 1. Introduction

**Figure 1.**
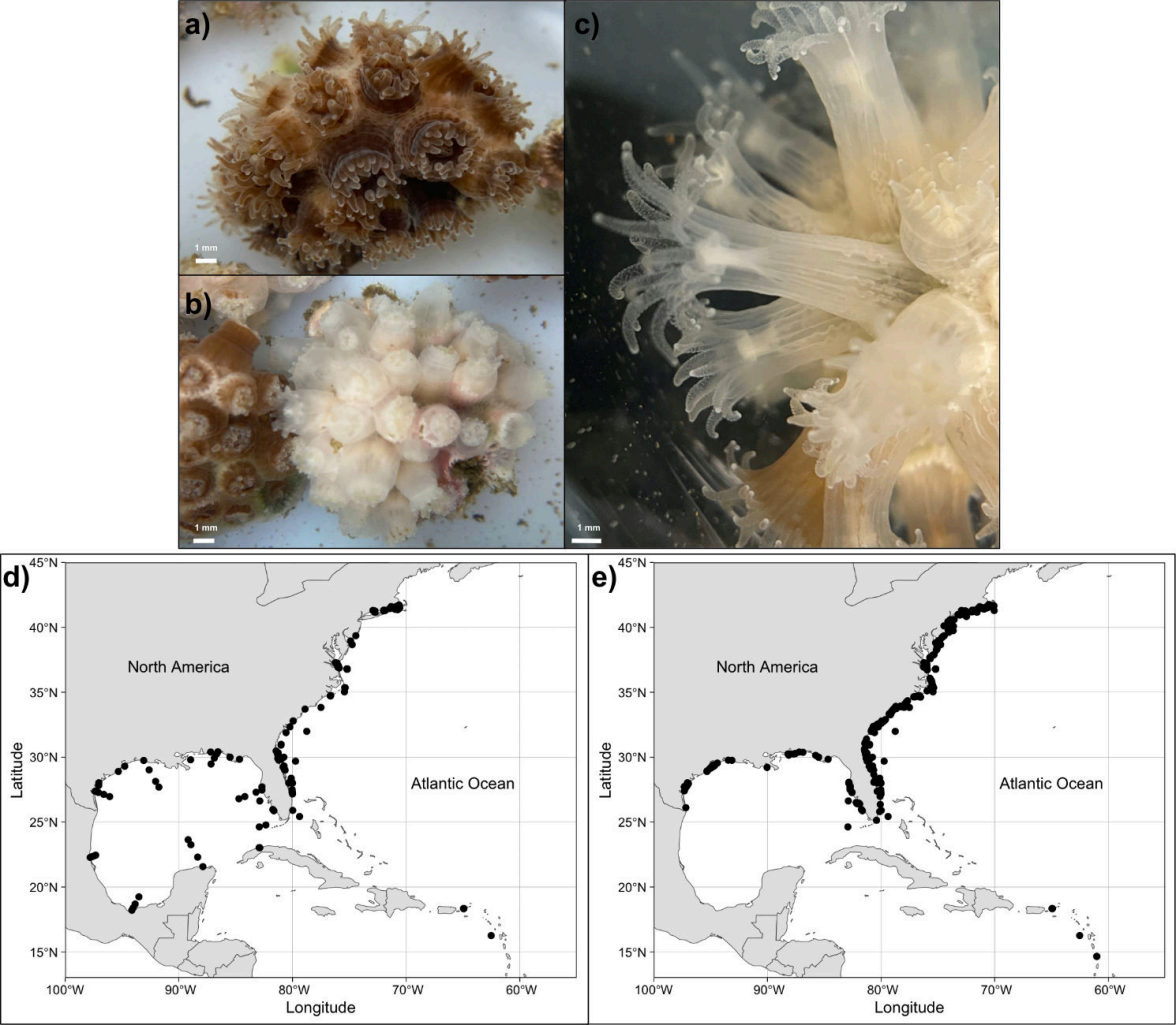
(a) Symbiotic and (b) aposymbiotic *Astrangia poculata* colonies. (c) Close-up of an aposymbiotic *A. poculata* polyp. Scale bar in (a–c) represents 1 mm. (d) Ocean Biodiversity Information System (OBIS) and (e) Global Biodiversity Information Facility (GBIF) data on *A. poculata* distribution in the western Atlantic Ocean. Photos by Chloé Gilligan and Hollie Putnam.

The northern star coral, *Astrangia poculata* (formerly *Astrangia danae* and *Astrangia astreiformis*; [1]), is a temperate scleractinian coral inhabiting coastal waters along the western Atlantic from the Gulf of Mexico to Massachusetts (figure 1). Unlike tropical corals, which form obligate symbiosis, *A. poculata* is facultatively symbiotic, existing on a spectrum from many (previously described as symbiotic; brown morphotype) to few symbionts (previously described as ‘aposymbiotic’; white morphotype [1]). These corals form calcium carbonate skeletons in all symbiotic states [2] and withstand extreme conditions that can trigger quiescence responses [3]. This unique set of biological aspects highlights the broad utility of this temperate coral for addressing research questions ranging from ecological to cellular scale questions, and here we leverage this growing body of literature to synthesize the state of knowledge for *A. poculata*.

Research surrounding *A. poculata* is rapidly expanding, with approximately 50% of *A. poculata* literature published in the last two decades and its recent designation in 2021 as the ‘Rhode Island State Coral’ (Rhode Island House Bill 2021-H 5415, Rhode Island Senate Bill 2021-S 0067). Despite growing focus on fundamental aspects of *A. poculata*’s biology and ecology, it remains understudied relative to its geographical range and experimental value. To address this, the present review synthesizes existing literature (peer-reviewed and published) as of January 2025 on *A. poculata* into six focal areas: geographic range, habitat and ecology, symbiosis, life history, microbiome and genomics and transcriptomics. We also point to pre-printed studies as indicators of research directions. While one lens of viewing *A. poculata* could be as a model for tropical corals, an in-depth exploration of *A. poculata* as a model organism is beyond the scope of this review. Here, we overview and synthesize ongoing *A. poculata* research from the lens of the broad capacity of this system for testing hypotheses of ecology, symbiosis, population genetics, biomineralization and resilience to environmental perturbations. Our goal is to describe how this rapidly growing body of literature around *A. poculata* can contribute to a deeper understanding of the resilience of marine species to environmental change.

## 2. Literature review

**Figure 2.**
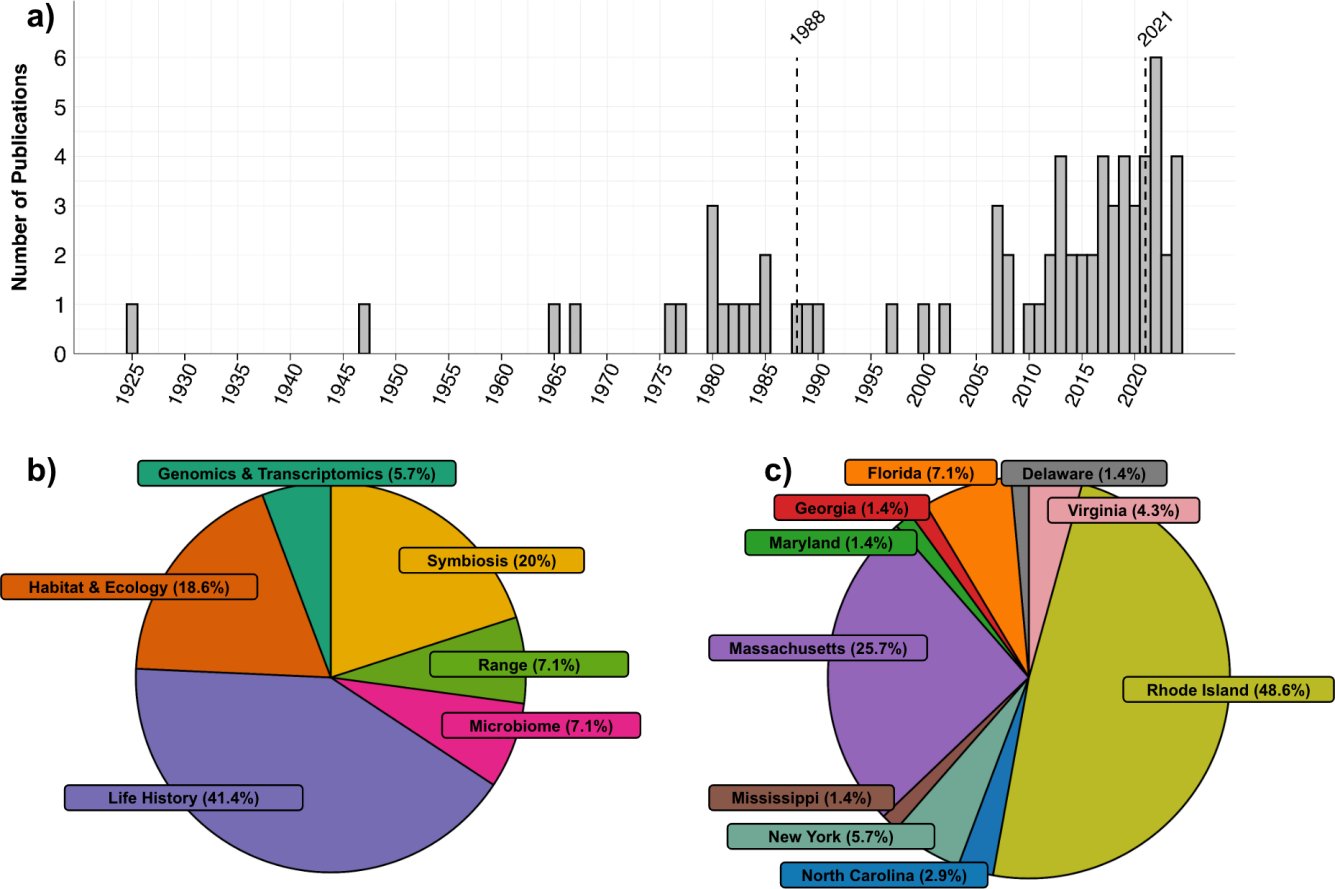
(a) Frequency of published *Astrangia poculata* literature over time. Publication numbers reflect peer-reviewed research articles; only publications prior to January 2025 are included. Dotted lines indicate important dates in *A. poculata* history. The name of the species changed from *Astrangia danae* to *A. poculata* in 1988 [1]. *Astrangia poculata* became the Rhode Island State Coral in 2021. (b) Percentages of the primary focal areas from published *A. poculata* literature. (c) Percentages of locations where *A. poculata* was studied.

A scientific literature review was conducted to assess the current state of knowledge for *A. poculata*, focused on peer-reviewed articles from the past century (1925–2025) and observational reports from Information Systems and Facilities (1903–2025). Using major search engines (Google Scholar, Web of Science), literature was compiled using search terms including the current (*A. poculata*) and former scientific names (*A. danae; A. astreiformis*). Collected literature was tabulated (electronic supplementary material, table S1), documenting the publication year and study location for each article. Seventy peer-reviewed research articles on *A. poculata* were identified beginning in 1925, with approximately 50% published since 2002 (figure 2a). We summarized the literature into six focal areas*—*geographic range, habitat and ecology, symbiosis, life history, microbiome and genomics and transcriptomics—based on accompanying key words from the manuscripts. Life history was most comprehensively covered (41.4% of publications, figure 2b), while genomics and transcriptomics had only four studies (5.7% of publications, figure 2b).

### (a) Geographical range

To enhance the understanding of *Astrangia poculata* distribution*,* observational data from the Ocean Biogeographic Information System [4] and Global Biodiversity Information Facility [5] were reviewed to complement the literature. The OBIS and GBIF datasets had 254 and 582 unique coordinates, respectively. OBIS data (figure 1d) spanned observations from 1903 to 1993, while GBIF data (figure 1e) ranged from 1968 to 2024. Nine observations were shared between the OBIS and GBIF datasets, underscoring their uniqueness and the advantage of evaluating both. The majority of observations occurred in the Northwest Atlantic, while two observations (Portugal and the Pacific coast of Mexico) fell outside of this range. Such occurrences may be attributed to dispersal by rafting on other organisms [6] or debris [7] or to species identification errors. In the United States, *A. poculata* colonies have been studied across nine states, with a significant proportion of research (approx. 75%) concentrated in the most northern range in Rhode Island and Massachusetts (figures 1d,e and 2c). This geographical bias implies limitations in generalizing findings exclusively from northern populations. For instance, in tropical symbiotic corals, physiological responses [8], reproductive strategies [9] and ecological interactions [10] can vary across latitudes or with local conditions. Therefore, research on the basic biology across the full range of *A. poculata* at the levels of cells to communities is vital to distinguish local from generalizable conclusions.

### (b) Habitat and ecology

*Astrangia poculata* forms encrusting colonies on hard, low silt-clay substrates [1,11–13] and has been observed on structures like sunken ships or wind turbine foundations [14,15]. It tolerates temperatures ranging from −1°C to 27°C [2]. Using temperature and growth data, Dimond *et al.* [16] demonstrated that low temperatures limit its northern distribution, with winter tissue loss hindering net growth beyond Cape Cod, Massachusetts [16]. Winter quiescence, marked by arrested physiological and metabolic processes, further supports this limitation [3] in Rhode Island populations. Given the facultative symbiotic nature, *A. poculata* requires light for symbiotic autotrophy or heterotrophic nutrients for symbiont and host growth [17,18]; thus, these factors should shape distribution. Further, as *A. poculata* biomineralizes, carbonate chemistry will influence the capacity to settle and grow through early life stages which are more susceptible to competition and prediction [19]. Thus, additional environmental factors that drive the range should be clarified.

*Astrangia poculata* hosts various annelids, amphipods and sponges [1,20] and has been documented as an epibiont on loggerhead sea turtles [6], hermit crabs [21] and bivalves [22]. Its co-occurrence with various organisms suggests a role in supporting biodiversity. However, no significant correlation was found between fish abundance and *A. poculata* coverage on artificial reefs in the Mid-Atlantic Bight, although this study focused on sea whip corals [14]. This suggests that its influence on fish communities is negligible, indirect or context-dependent. Given that *A. poculata* holds space on the benthos, creates a three-dimensional structure, and has a role in nutrient cycling, it is important to consider this coral within the framework of community ecology, as its presence may influence local species assemblages through habitat modification or trophic interactions.

### (c) Symbiosis

*Astrangia poculata* is facultatively symbiotic, maintaining symbiont densities ranging from 10^4^ to 10^7^ cells cm^−2^ [23]. Colonies with symbiont densities ≤ 10^5^ cells cm^−2^ appear visibly pale and have previously been defined and discussed as ‘aposymbiotic’ in the *A. poculata* literature [24,25]. When we use the term ‘aposymbiotic’ in this review, we do so for clarity with the papers we are discussing, but we strongly reinforce that these corals previously designated ‘aposymbiotic’ are not without symbionts entirely (e.g. a primary paper cited as ‘aposymbiotic’ corals quantifies these as 10^4^−10^5^ cell cm^−2^; [23]). *Breviolum psygmophilum* (formerly *Symbiodinium psygmophilum*, clade B [26,27]) is *A. poculata*’s dominant algal endosymbiont. The ability of *B. psygmophilum* to exhibit wide temperature tolerance and to rapidly recover photochemical efficiency under stress [28] likely contributes to *A. poculata*’s success across a wide latitudinal gradient.

When in symbiosis with *B. psygmophilum*, *A. poculata* displays enhanced survivorship, wound healing and immune response [18,29,30]. Wounded symbiotic colonies demonstrated more rapid rates of tissue recovery compared with ‘aposymbiotic’ colonies, with a greater proportion of symbiotic colonies developing new tissue at lesion sites [18,29,30]. Constitutive immunity in *A. poculata* is associated with high symbiont densities, with positive correlation between symbiont density and both melanin concentration and catalase activity [31,32]. Higher symbiotic densities, which elevate photogenerated oxygen and drive reactive oxygen species production, could require increased activity in immune-related pathways in the host in topical corals (reviewed in [33]). Alternatively, greater symbiont density may supply more photosynthates, enabling the coral to allocate additional resources to immune functions (as hypothesized by [31]), yet no translocation studies have been conducted in *A. poculata* to date. The relationship between symbiosis, immunity and wound healing warrants further investigation, particularly in colonies exposed to stressors (i.e. temperature, disease), to determine whether symbionts provide immune-related advantages during periods of stress.

Symbionts can impose costs on the host under stress. One example is cold stress in Rhode Island populations, where symbionts stop photosynthesizing below 15°C and become heterotrophic, sequestering resources from the host [2,16,25]. The decline in symbiont density and increased symbiont expulsion observed during New England winters further suggests that maintaining symbionts can impose metabolic costs, leading to temporary or seasonal parasitism [25]. Another example is the cost of hosting symbionts when autotrophic and heterotrophic energy sources are removed due to sediment exposure and heterotrophic starvation. Symbiotic colonies exposed to sediment and starvation were more susceptible to mucous cell loss and had higher respiratory demands than ‘aposymbiotic’ colonies [34]. Symbiosis emerges as a core facet of *A. poculata*’s biology that needs to be further investigated; given its relevance in the biological processes of *A. poculata*, symbiosis will be further discussed in the context of other focal areas as follows.

### (d) Life history

#### (i) Reproduction

*Astrangia poculata* is a gonochoric broadcast spawner, releasing eggs and sperm for external fertilization [35]. It was the first coral species for which histological methods were applied for systematic documentation of gametogenesis and inferred spawning, along with spawning induced in the laboratory [35]. Germ cells develop in the mesenterial mesoglea, forming spermaries and oocytes four months before spawning (March to July–August) [35]. Successful fertilization produces swimming larvae within 12−15 h ([35] personal observation by JA, 2021–2022). Eggs lack symbionts, indicating horizontal (from the environment) symbiont uptake [35]. Sperm motility is controlled by signalling pathways involving cytosolic alkalinization, soluble adenylyl cyclase and protein kinases, with these pathways exhibiting homology across gonochoric cnidarians, implying functional conservation [36]. The symbiotic state does not primarily determine sperm release, reflecting the contrasting patterns of the contributions of autotrophy compared with heterotrophy in *A. poculata* (i.e. no relationship between symbiont density and energy content of tissue [35,37]) compared with tropical corals, where there are positive relationships between symbiont density and energy reserves [38]. Gamete provisioning in symbiotic and ‘aposymbiotic’ colonies remains largely unexplored. In tropical corals, autotrophy typically provides carbon as the allocated energy source for gametogenesis [39]. However, it is unknown how carbon acquired via autotrophy versus heterotrophy provisions gametes, which warrants further investigation using isotopic labelling techniques. There is no current published literature regarding larval survival or development (but see pre-print [40]), and the life cycle of *A. poculata* has not been closed in published literature. Further research on development, settlement and survivorship is crucial for biological, genomic and ecological insights.

#### (ii) Growth and calcification

Growth in *A. poculata* is typically measured by polyp count, reflecting three-dimensional colony growth [24]. Growth varies seasonally, with growth from May–December and cessation from December–April in Rhode Island [3,24]. During this period, polyp loss due to tissue thinning and metabolic quiescence makes colonies susceptible to overgrowth by benthic colonizers [24]. Growth can also be examined as tissue biomass and energetic content of the tissue (e.g. carbohydrates, lipids, proteins). Lipid content, chlorophyll a and c2, and photosynthesis are lowest following winter (May), with high delta ^15^N content in host and symbiont indicating depleted lipid reserves, despite increased heterotrophy, which cannot fully compensate [41]. Growth persists in fed corals under sediment stress but halts in starved corals [34].

Like other calcifying scleractinian corals, *A. poculata* extracts dissolved inorganic carbon and calcium from seawater to form calcium carbonate skeletons [42]. It maintains a high pH in calcifying fluids through a fixed external–internal H^+^ ratio [43] and uses intracellular calcification via crystal-packed vesicles for skeletogenesis [44]. Calcification increases with light and temperatures [2,45,46], showing linear calcification from 6.5–27°C and stimulation at light intensities >40 µE in m^−2^ s^−1^ above 6.5°C [2]. While elevated pCO_2_ reduces calcification [43,47], the combination of elevated pCO_2_ and nutrient enrichment mitigates these decreases, likely from stimulated photosynthesis and carbon translocation [47]. Reduced calcification rates in female *A. poculata* exposed to high pCO_2_, compared with males at high pCO_2_ and males and females grown at ambient pCO_2_, illustrate the reproductive energetic consequences [48]. Although these studies offer valuable insights into *A. poculata* growth and calcification, they focus exclusively on corals from the northernmost range, limiting their generalizability to other populations and underscoring the need for range-wide research to assess resilience to climate change.

A research need in *A. poculata* and tropical corals is understanding how the symbiotic state influences calcification from biological and materials science perspectives. Symbiont presence explained 65% of the variance in the strontium−calcium ratio, a palaeothermometer used to estimate sea surface temperatures, in coral skeletons [49], suggesting that ‘aposymbiotic’ *A. poculata* skeletons are more reliable for past temperature estimates. Further, symbiotic colonies exhibited higher amounts of specific, uncharacterized metabolites and C_13_ isotope enrichment, indicative of differential metabolic pathway usage and greater incorporation of photosynthetically fixed carbon [50,51]. Microscale calcification roughness is also dependent on symbiont density and the interactions with temperature; at 27°C, ‘aposymbiotic’ colonies had rougher surfaces than symbiotic colonies, indicative of inhibited calcification, while the trend was reversed at 15°C, with symbiotic colonies having rougher surfaces [46]. This suggests that secondary infilling or thickening deposits are enhanced by the presence of symbionts, which could be attributed to enhanced carbon translocation providing the energetic substrate to fuel calcification via generation of critical skeletal organic matrix proteins such as coral acid-rich proteins, the active control of pH within the calcifying space or the active transport of essential ions [52]. This pattern is likely reversed at colder temperatures due to reduced rates of photosynthesis and temperature-facilitated dissolution of the aragonite skeleton [46]. This symbiont and light-enhanced calcification found in *A. poculata* reinforces the need for further study of symbiont modulation of physiological and cellular processes.

#### (iii) Autotrophy, heterotrophy and metabolism

*Astrangia poculata* primarily relies on heterotrophy, consuming marine organisms (amphipods, copepods, ctenophores, etc.) and inert materials [53]. While autotrophy supports carbon and nutrient translocation in *A. poculata*, it cannot fully supplement the coral’s nutritional needs without biomass loss in Rhode Island populations [17,54,55]. Relative reliance on autotrophy and heterotrophy varies by season, with higher autotrophy rates in spring and higher heterotrophy rates in winter [41]. Notably, these seasonal variations were driven by the host’s carbon and nitrogen acquisition, with symbiont contributions remaining constant year-round [41].

Reliance on heterotrophy in *A. poculata* increases susceptibility to pollutant and contaminant ingestion. *Astrangia poculata* preferentially ingests unfouled rather than biofouled plastics [56], suggesting chemosensing capacity to discriminate between unfouled versus biofouled materials. Further, *A. poculata* ingested plastic spheres and subsequently did not ingest brine shrimp eggs [57], indicating ingestion as an avenue for the introduction of potentially polluted materials. Higher heterotrophic feeding in winter is linked to increased heavy metal accumulation (lead, zinc, chromium, cadmium) in *A. poculata* tissue [54]. These findings underscore the complexity of *A. poculata*’s nutritional strategies and their implications for ecological resilience, as reliance on autotrophy and heterotrophy influences nutrient acquisition and potential susceptibility to environmental contaminants.

Photosynthesis and respiration are vital physiological indicators for assessing coral metabolism. Across a temperature gradient (11.5–23°C), *A. poculata*’s respiration rates remain consistent, demonstrating metabolic stability [2]. At 6.5°C, minimal metabolic activity occurred, indicating quiescence onset [2]. A curvilinear relationship was observed between photosynthetic rates and light intensity, with symbiont photosynthetic capabilities reaching their maximum around a light intensity of 400 µE in m^−2^ s^−1^ [2]. Additionally, photosynthesis and respiration rates increased when corals were fed, underscoring metabolic responsiveness to nutrient availability [17,45]).

Most studies on *A. poculata* respiration and photosynthesis have focused on northern populations, but Aichelman *et al.* [58] compared rates from Rhode Island and Virginia colonies. Rhode Island colonies exhibited higher respiration rates, indicating countergradient variation, which occurs when a cold-adapted population exhibits an elevated phenotype compared with a warm-adapted population [58]. Conversely, Virginia colonies had a higher thermal optimum (*T*_opt_) for respiration, with *T*_opt_ 3.8°C higher in symbiotic and 6.9°C higher in ‘aposymbiotic’ colonies compared with Rhode Island counterparts [58]. These findings suggest local adaptation, which may enhance resilience under ocean warming. Studying facultatively symbiotic colonies from multiple locations will provide powerful inferences regarding acclimatization/adaptation, nutritional strategy and seasonal energy modulation.

#### (iv) Nitrogen cycling

Nitrogen cycling is the process by which nitrogen is transformed, utilized and exchanged within the coral holobiont (i.e. the coral host and associated microorganisms) and between the coral and its environment [59]. Cycling includes inorganic (e.g. ammonium and nitrate) and organic (e.g. amino acids) nitrogen acquired through symbiont-mediated processes (if symbiotic) and heterotrophy (if aposymbiotic). *Astrangia poculata* exhibits different nitrogen assimilation depending on symbiotic state [60]. Residual symbionts are the primary explanation for nitrogen uptake as ‘aposymbiotic’ colonies often retain substantial symbiont densities (10^4^–10^5^ cells cm^–2^; [23]), and DiRoberts *et al.* [60] did not measure symbiont density in their study. ‘Aposymbiotic’ and symbiotic *A. poculata* can assimilate ammonium more efficiently than nitrate, but only fed symbiotic colonies substantially assimilate nitrate [60], suggesting that nitrogen uptake is energetically costly and requires energy input from autotrophic or heterotrophic sources. ‘Aposymbiotic’ colonies assimilate ammonium when fed, indicating that environmental nutrients are largely inaccessible to the holobiont when no autotrophic or heterotrophic inputs are present [60]. Mechanisms by which truly aposymbiotic colonies could assimilate inorganic nitrogen remain untested in *A. poculata*. One possibility is microbiome-mediated nutrient transformations that enhance nitrogen availability to the host, which has been shown in tropical corals [61]. Alternatively, assimilation could occur through host-derived pathways (e.g. glutamine synthetase–glutamate synthase pathway), as both symbiont and coral cells have demonstrated the capacity to fix nitrogen from ammonium-enriched seawater [62]. Additionally, symbiotic colonies excrete less nitrogen overall, due to symbiont-mediated uptake [17]. This flexibility in nitrogen acquisition and usage in *A. poculata* and the adaptability to different nitrogen sources suggest resilience in environments where nutrients fluctuate, helping *A. poculata* survive in temperate ecosystems with variable nutrient availability.

### (e) Microbiome

The microbiome of *A. poculata* undergoes dynamic changes linked to seasonal shifts and symbiotic state. Microbial composition does not differ between ‘aposymbiotic’ and symbiotic colonies but does change significantly across seasons [23,63,64]. As corals enter quiescence, pathogens and copiotrophic bacteria are shed, while nitrification-associated microbes increase, potentially supplying nitrate to colonies during non-feeding quiescent periods [64]. The microbiome’s significant restructuring suggests its involvement in initiating and sustaining quiescence [64], or alternatively, a strong response to host cellular and physiological changes during these periods. The microbiome of *A. poculata*, assessed within a symbiotic context, shows significant microbiome alterations due to antibiotic-induced disturbance [65]. Although the microbiome rapidly re-established itself within two weeks, symbiotic colonies exhibited more similar microbial compositions during recovery compared with ‘aposymbiotic’ colonies, suggesting that algal symbionts influence the microbiome re-establishment process, potentially by providing nutrients and signalling molecules [65]. The microbiome of *A. poculata* may also contribute to nitrogen cycling, particularly during winter when microbes involved in ammonia oxidation, nitrification and nitrogen fixation increase, suggesting a possible role for the microbiome in nitrogen acquisition when the coral enters a quiescent, non-feeding state [64]. The dynamic restructuring of *A. poculata*’s microbiome across seasons and during disturbance recovery underscores its vital role in many aspects of coral physiology.

### (f) Genomics and transcriptomics

Understanding the biology of *A. poculata* and its mechanisms of adaptation and acclimatization is essential, particularly in the face of environmental stressors. Research has focused on transcriptomic responses in ‘aposymbiotic’ and symbiotic *A. poculata* under thermal and disease stress. Regardless of symbiotic state, cold stress (4–6°C) elicited a stronger transcriptomic response than heat stress (30–31°C) [66,67]. Under heat stress, symbiotic colonies displayed higher expression plasticity in genes related to cell cycle pathways involved in symbiont growth, but a dampened environmental stress response (i.e. genes related to stress responses), leading the authors to suggest that photosymbiosis may suppress the host’s stress response to environmental challenges [67]. In response to the pathogen *Vibrio coralliilyticus*, ‘aposymbiotic’ colonies upregulated genes related to cilia construction and movement, while symbiotic colonies upregulated immune genes [68]. The authors posit that ‘aposymbiotic’ colonies may be triggering less energy-intensive preventative defences, whereas symbiotic colonies appear to be activating a more energetic-costly immune response [68], which is in line with the potential for higher carbon translocation and lipid reserves in symbiotic corals that could be used as energy substrates.

Population genetics of *A. poculata* and *B. psygmophilum* reveal important insights into the host’s adaptive responses to environmental pressures. Aichelman & Barshis [69] conducted the only study to date examining genetic variations between host and symbiont populations in Rhode Island and Virginia, identifying potential loci as adaptive signatures of the two populations. Contrasting dynamics of population connectivity and dispersal were inferred between host and symbiont populations. Differentiation between Rhode Island and Virginia host populations was driven by putatively adaptive loci associated with stress response genes, while neutral population differentiation was observed in the symbiont populations [69]. These results suggest that the coral host is responding to and adapting to environmental pressures, while the symbiont may not be experiencing the same selective pressures.

Preprints on *A. poculata* genomics and transcriptomics, although not included in our analysis due to lack of peer review, provide insights into the direction of the field. Recently, assembly and annotation of the *A. poculata* genome highlighted expanded gene families (relative to tropical coral *Acropora millepora*) related to sleep promotion, dormancy, transposable elements and histone proteins, providing compelling mechanistic evidence of *A. poculata* plasticity and adaptation [70]. Additionally, gene knockdown and CRISPR-mediated gene knock-in have been applied to inhibit fibroblast growth factor A1 and facilitate the insertion of fluorescent tags into marker genes, demonstrating *A. poculata* as a tractable experimental organism for developmental research [40]. The growing application of -omic methodologies and molecular techniques to *A. poculata* reflects the increasing interest in and potential for rapidly advancing our understanding of the molecular mechanisms governing stress responses, development and adaptation.

## 3. Outstanding research questions and recommendations

**Figure 3.**
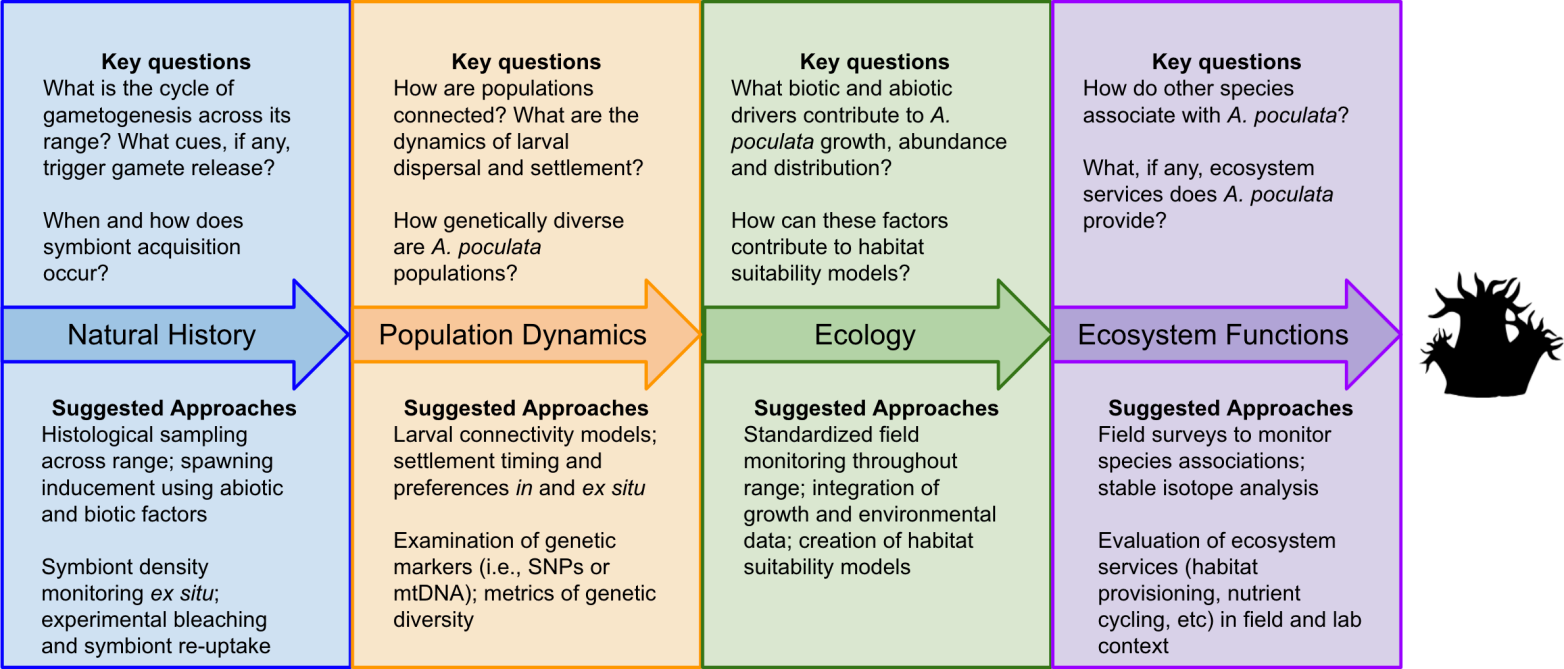
Outstanding key questions and suggested approaches to *Astrangia poculata* research. Coral silhouette from https://github.com/sPuntinG/Coral_stuff/tree/main.

Our literature review highlights future research opportunities that are fundamental for advancing our understanding of *A. poculata* biology, ecology and ecosystem roles (figure 3) including (i) natural history (reproduction and symbiont acquisition); (ii) population dynamics (structure and connectivity); (iii) ecology (habitat preferences and species interactions); and (iv) ecosystem functions and services (trophic interactions and biogeochemistry and ecosystem services). While some of these research areas may appear primarily foundational, not only will they clarify knowledge across environments and populations but they will also provide the essential building blocks for more transformative scientific advances. Similarly, exploring *A. poculata*’s ecological roles and contributions to ecosystem function can uncover species interactions, biogeochemical processes and elucidate the abiotic and biotic factors shaping its distribution.

### (a) Natural history

#### (i) Reproduction

Despite early studies on *A. poculata* gametogenesis, understanding of *in situ* reproductive dynamics remains limited, with no wild spawning data available to date. *In situ* monitoring during spawning periods is challenging due to unknown spawning cues and egg characteristics (approx. 75–100 μm, negatively buoyant; personal observation by JA, 2021-2022). We recommend comprehensive histological monitoring of gametogenesis across *A. poculata*’s range to determine reproductive maturity timing and how environmental factors associated with latitude may influence gamete maturation, abundance and parental provisioning. Additionally, sampling different-sized colonies throughout the year can provide insights into gametogenesis onset and development. Fine-scale collections (i.e. days to weeks) and histological data around expected spawning times would also be conducive to capturing precise dates of gamete release.

#### (ii) Symbiont acquisition

Symbiotic and ‘aposymbiotic’ *A. poculata* colonies are present throughout the entirety of their range, but it is unknown how or when larvae, recruits, or adult colonies acquire symbionts. To better understand the symbiont acquisition mechanism and timing in *A. poculata*, larvae and recruits (i.e. smallest detectable colonies) should be monitored *in situ* and *ex situ* over time to track symbiont densities. Such monitoring will be beneficial throughout the range of *A. poculata* or under experimental conditions, as northern populations may be less receptive to symbiont uptake and maintenance than colonies in warmer conditions [25]. Further, experimental bleaching of symbiotic *A. poculata* colonies would provide the opportunity to observe the dynamics of re-colonization by the symbionts in the newly ‘aposymbiotic’ colonies under different environmental parameters.

### (b) Population dynamics

#### (i) Population connectivity

The connectivity of coral populations is facilitated by larval dispersal and settlement [71]. While field spawning times are still unknown, *ex situ* generation of larvae can be used to generate propagules to examine larval energetics, as well as larval pelagic duration and behaviour under various environmental conditions. By combining biological (reproductive output and timing, larval pelagic time and behaviour, etc.) and biophysical (wind speed and direction, ocean conditions, etc.) factors [72], connectivity models can be generated for testing as field spawning data become available. Another major gap in our understanding of population connectivity is settlement preferences and timing. To date, no peer-reviewed literature has documented *A. poculata* settlement *ex* or *in situ*. To assess competency and settlement preferences, settlement assays should be done *ex situ* by providing larvae with various substrates (i.e. rocks, shells, glass slides, aragonite plugs, ceramic tiles, etc.) [73]. Alternatively, settlement tiles should be deployed monthly or biweekly *in situ* in locations with high abundance of *A. poculata* to assess larval settlement timing, location and preference [74].

#### (ii) Population structure

Investigations into the population structures of *A. poculata* have been limited [69], and it is unknown if there are genetically diverse populations (limited gene flow, differing life-history characteristics and independent evolutionary trajectories) or metapopulations (some level of connectivity, gene flow and life-history traits among populations) throughout its range. We recommend a concerted sampling effort through the range of *A. poculata* to create high-resolution population genetic resources [75].

### (c) Ecology

Questions remain about determinants of *A. poculata* distribution, habitat preferences and species interactions. Previous investigations have predominantly focused on temperature and its relationship to *A. poculata* growth, with studies confined to the species’ northern distribution [16]. To gain a more holistic understanding of the environmental drivers governing the growth and calcification of *A. poculata*, we recommend integrating numerous abiotic measurements (i.e. temperature, flow, pH, salinity, light, nutrients, carbonate chemistry, etc.) with growth and calcification data across its range to create habitat suitability models, which can be utilized to predict the distribution of *A. poculata* along its extensive range. Habitat suitability models specific to *A. poculata* can also forecast potential impacts of climate change on *A. poculata* distribution by projecting how suitable habitats might shift under different climate scenarios [76].

We recommend establishing standardized population monitoring programmes throughout *A. poculata*’s range to track abundance and distributional changes over time. Observational information can illuminate the species’ range (figure 1d and e), but cannot differentiate areas of abundance at a fine scale. Field monitoring techniques should include quadrat sampling, transect sampling and photographic surveys and could be leveraged for genetic surveys as well. Sampling/surveys should ideally be conducted at multiple times throughout the year to account for potential changes in seasonal phenology [23,41,54]. Such standardized abundance information, habitat data and genetics collected over time will allow for availing analytical tools to be developed for understanding preferable conditions and predicting potential *A. poculata* distribution (e.g. [16]).

### (d) Ecosystem functions and services

#### (i) Trophic interactions

It is unknown what role *A. poculata* serves in larger marine ecosystem contexts or the significance of their interactions with other species. Field surveys around populations of *A. poculata* should be conducted to monitor how *A. poculata* associates with other taxa. Moreover, studies should focus on the role of *A. poculata* in the food web, which can provide insights into its influence on other species as a predator, prey or competitor. For instance, stable isotope analysis (e.g. C^13^, N^15^) can be employed to understand trophic position and feeding relationships of *A. poculata* [77]. Stomach content analyses of associated fauna may also reveal what species consume *A. poculata,* which can be incorporated into ecosystem models.

#### (ii) Biogeochemistry and ecosystem services

Foundational species or ecosystem engineers such as reef-building corals and seagrasses drive ecosystem functions and services such as biogeochemical cycling [78,79], primary production and generation of three-dimensional habitat [80] and coastal protection structure [80,81]. The role of *A. poculata* in a temperate reef setting is less well characterized. While *A. poculata* produces a skeleton [43] that contributes to benthic rugosity and internal symbiotic nutrient cycling is increasingly well reported [60] (see section above), the trophic state and interactions, as well as the implications for coastal ocean biogeochemistry, are not yet quantified. For example, the contribution of *A. poculata* to important functions such as net ecosystem calcification (the sum of calcium carbonate production and dissolution) and net ecosystem productivity (gross primary productivity minus total ecosystem respiration), which are critical on tropical coral reefs, are unknown on temperate reefs, where for example, the per cent cover of *A. poculata* has been reported as high as 9% [82]. Services like habitat provisioning, nutrient cycling, sediment stabilization or water filtration may be supported by *A. poculata* based on those of other corals [83] but this remains unknown. Once quantified with respect to *A. poculata*, these services can be considered with respect to economic valuation to describe the monetary value that they provide to human well-being.

## 4. Conclusions

With the accumulated knowledge and insight of 100 years of *A. poculata* studies generated by experts focused on this taxa and newer arrivals to the field, we have an ability to understand the biological and ecological significance of this experimental system and resolve knowledge gaps. Our systematic review of *A. poculata* literature indicates that future studies will benefit from continuing the transition from dichotomy of the symbiotic and ‘aposymbiotic’ categories and embracing the gradient of symbiotic density of ‘facultative photo-endosymbiotic’ nature (*sensu* [84]) to further elucidate the modulation of environmental responsiveness through symbiosis. Rapidly advancing sequencing and computational approaches paired with ecologically relevant spatio-temporal sampling will help to resolve the genetic and environmental factors contributing to *A. poculata* geographical distribution and community and ecosystem consequences. Researchers are now also poised for essential knowledge gap infilling, as well as deep insight into development and cellular physiology. Building from the work synthesized here, via open and inclusive engagement with the broader field of symbiosis, evo-devo, genetics and climate change research, and emphasis on data sharing and communication, will facilitate not only the closing of identified knowledge gaps but also the advancement of novel and interdisciplinary science around this dynamic system.

## Data Availability

All data and scripts for this manuscript are available from Zenodo [85]. Supplementary material is available online [86].
